# Derivation and Validation of Shock Index as a parameter for Predicting Long-term Prognosis in Patients with Acute Coronary Syndrome

**DOI:** 10.1038/s41598-017-12180-2

**Published:** 2017-09-20

**Authors:** Tongtong Yu, Chunyang Tian, Jia Song, Dongxu He, Zhijun Sun, Zhaoqing Sun

**Affiliations:** Department of Cardiology, Shengjing Hospital of China Medical University, Shenyang, Liaoning P.R. China

## Abstract

The objective of this study was to examine whether shock index (SI), defined by ratio of heart rate and systolic blood pressure, can predict long-term prognosis of acute coronary syndrome (ACS) in patients undergoing percutaneous coronary intervention (PCI) and to compare prognostic accuracy of SI with the Global Registry of Acute Coronary Events (GRACE) risk score. This study included individuals from 2 independent cohorts: derivation cohort (n = 2631) and validation cohort (n = 963). In the derivation cohort, we derived that higher admission SI was associated with a greater risk of long-term all-cause mortality [HR = 4.104, 95% CI 1.553 to 10.845, *p* = 0.004] after adjusting for covariates. We validated this finding in the validation cohort [HR = 10.091, 95% CI 2.205 to 46.187, *p* = 0.003]. Moreover, admission SI had similar performance to the GRACE score in determining all-cause mortality risk in both cohorts (derivation cohort, admission SI *vs*. GRACE, *z* = 1.919, *p* = 0.055; validation cohort, admission SI *vs*. GRACE, *z* = 1.039, *p* = 0.299). In conclusion, admission SI is an independent predictor of adverse outcome in ACS patients undergoing PCI, and can identify patients at high risk of death. SI and the GRACE score showed similar performance in predicting all-cause mortality, and SI is more readily obtained than the GRACE score.

## Introduction

Acute coronary syndrome (ACS) describes a spectrum of clinical conditions connected with myocardial ischemia and/or infarction, including unstable angina, ST-segment elevation myocardial infarction (STEMI), and non-ST-segment elevation myocardial infarction (NSTEMI)^1–4^. ACS frequently has a poor prognosis^1–4^, but percutaneous coronary intervention (PCI), which is now widely available, can improve the prognosis^1–4^. However, there are some ACS patients who will remain at risk of adverse cardiac events even after PCI^1–4^. Early risk stratification can help to identify these high-risk patients to promote appropriate clinical treatment with close follow-up. The Global Registry of Acute Coronary Events (GRACE) score is an important prognostic tool in ACS. It can help to identify those patients who are at highest risk of in-hospital^5^, 6-month^6^ and even long-term^7^ (up to 4 years) mortality post ACS. However, there are some barriers to routine use of the GRACE score. Clinicians may lack access to the software (“app”) necessary to calculate the score. Furthermore, calculation of the GRACE score must be delayed until key laboratory values are available.

The shock index (SI) is determined by the ratio of heart rate and systolic blood pressure, usually measured on admission and before later interventions^8–20^. The SI was originally employed to evaluate hemorrhage and acute circulatory failure^8,9^. Although SI is very easily influenced by the patient status and medication treatment, it can be also very easily obtained at the bedside, allowing risk assessment to be completed as early and as quickly as possible. Recently its application has since expanded to other critical care settings including trauma, surgery, and sepsis^10,11^. Other studies have also shown that the SI is a useful index for rapid risk assessment in acute myocardial infarction (AMI)^12–20^. SI can predict short-term adverse outcomes in patients with STEMI^12^, and it is an independent predictor of short-term mortality^13–15^, long-term mortality^15–17^, microvascular damage^17^, and extent of myocardial injury^18^ in STEMI patients undergoing PCI. The association between SI and greater in-hospital mortality risk in patients with NSTEMI has also been confirmed^19^, and elevated SI also correlates with a poorer 5-year prognosis in patients with AMI undergoing PCI^20^. In most of these studies, SI values of<0.7 are considered normal^12,14,16,18,19^. We hypothesized that SI could predict long-term prognosis in ACS patients undergoing PCI and that it can identify those patients who are at high risk of adverse cardiac events. To prove this hypothesis, we first derived that the admission SI was useful for predicting long-term prognosis in ACS after PCI in a retrospective cohort; we then validated the result in another, independent, prospective cohort. Furthermore, we compared the prognostic performance of admission SI with that of the GRACE score in both cohorts.

## Results

### Description of the Derivation and Validation Cohorts

There were 2631 patients in the derivation cohort and 963 patients in the validation cohort. The distribution of basic characteristics for the two cohorts is shown in Table 1. Compared with the derivation cohort, patients in the validation cohort had higher LVEF and lower GRACE score, a lower prevalence of diabetes mellitus and three-vessel disease, and less frequent use of intra-aortic balloon pump, angiotensin-converting enzyme inhibitors/angiotensin receptor blockers, and beta-blockers. On the other hand, the validation cohort had a higher prevalence of dyslipidemia, history of MI, prior PCI, and STEMI and more frequent use of glycoprotein IIb/IIIa inhibitor, aspirin, clopidogrel, and statins. Baseline characteristics such as age and sex distributions were comparable in the two cohorts (Table 1).

**Table 1:** Baseline Characteristics of the Derivation and Validation Cohorts, median (IQR), or N (%), or means±SD.

Variable	Derivation Cohort, n = 2631	Validation Cohort, n = 963	*P* Value
Demographics			
Age, yrs	61.8 ± 11.6	61.3 ± 11.3	0.221
Female	825 (31.4)	278 (28.9)	0.152
Medical history			
History of Diabetes Mellitus	955 (36.3)	296 (30.7)	0.002
History of Hypertension	1488 (56.6)	550 (57.1)	0.765
History of Dyslipidemia	1734 (65.9)	680 (70.6)	0.008
Current/recent smoker	1360 (51.7)	499 (51.8)	0.947
History of renal dysfunction	212 (8.1)	78 (8.1)	0.967
History of MI	186 (7.1)	91 (9.4)	0.018
Prior PCI	205 (7.8)	100 (10.4)	0.014
Prior peripheral arterial disease	26 (1.0)	15 (1.6)	0.155
Presentation			
SBP on admission, mm Hg	134.4 ± 22.7	135.7 ± 22.6	0.136
Heart rate on admission, beats/min	75.2 ± 14.2	75.3 ± 14.0	0.221
LVEF, %	57.0 ± 9.6	58.6 ± 8.5	<0.001
SI	0.58 ± 0.15	0.57 ± 0.14	0.298
GRACE	130.9 ± 35.3	119.3 ± 34.5	<0.001
Diagnosis on admission			0.028
Unstable Angia	776 (29.5)	302 (31.4)	
NSTEMI	869 (33.0)	273 (28.3)	
STEMI	986 (37.5)	388 (40.3)	
Troponin-I on admission, ng/mL	0.71 (0.01, 17.67)	0.67 (0.01, 21.00)	0.900
PCI details			
Left main disease	249 (9.5)	84 (8.7)	0.497
Three-vessel disease	806 (30.6)	247 (25.6)	0.004
Intra-aortic Balloon Pump	135 (5.1)	25 (2.6)	0.001
TIMI flow grade 3 post PCI	2622 (99.7)	958 (99.5)	0.450
Use of glycoprotein IIb/IIIa inhibitor	827 (31.4)	376 (39.0)	<0.001
Medical treatment at discharge			
Aspirin	2532 (96.2)	955 (99.2)	<0.001
Clopidogrel	2511 (95.4)	938 (97.4)	0.008
Ticagrelor	32 (1.2)	12 (1.2)	0.943
Statin	2491 (94.7)	947 (98.3)	<0.001
ACEI / ARBs	1509 (57.4)	401 (41.6)	<0.001
Beta-blockers	1414 (53.7)	386 (40.1)	<0.001

MI, myocardial infarction; bpm, beats per minute; LVEF, left ventricular ejection fraction; h, hour; PCI, percutaneous coronary intervention; ACEI / ARBs, Angiotensin-converting enzyme inhibitors / Angiotensin receptor blockers.

### Prognostic Value of Shock Index in the Derivation Cohort

During an average follow-up period of 32 months, there were 80 events (3.0%) of all-cause mortality in the derivation cohort. Significant predictors of all-cause mortality at univariate analysis included admission SI, age, history of MI, prior PCI, prior peripheral arterial disease, LVEF, troponin-I on admission, three-vessel disease, intra-aortic balloon pump, TIMI flow grade 3 post PCI, and discharge prescription of beta-blockers and angiotensin-converting enzyme inhibitors/angiotensin receptor blockers (*p* < 0.05; Appendix S1, Table 2). After adjusting for covariates, higher admission SI continued to show significant positive correlation with the long-term all-cause mortality rate [HR = 4.104, 95% CI = 1.553–10.845, *p* = 0.004] (Table 2).

**Table 2:** Effects of admission SI and GRACE on the outcome in Univariate and Multivariate of the Derivation and Validation Cohorts.

	Univariate Analysis	Multivariate Analysis		
	HR(95%CI)	P	HR(95%CI)	P
Derivation Cohort				
SI	6.364 (2.802–14.452)	<0.001	4.104 (1.553–10.845)	0.004 ^a^
GRACE	1.018 (1.013–1.024)	<0.001		
Validation Cohort				
SI	12.848 (2.327–70.945)	0.003	10.091 (2.205–46.187)	0.003 ^b^
GRACE	1.023 (1.013–1.033)	<0.001		

^a^Adjusted for age, history of MI, prior PCI, prior peripheral arterial disease, LVEF, Troponin-I on admission, Three-vessel disease, Intra-aortic balloon pump, TIMI flow grade 3 post PCI and discharge prescription of beta-blockers, Angiotensin-converting enzyme inhibitors / Angiotensin receptor blockers;

^b^Adjusted for age, LVEF, diagnosis on admission and TIMI flow grade 3 post PCI.

The ROC-AUC of admission SI and GRACE for predicting all-cause mortality in the derivation cohort were 0.619 (95% CI 0.600 to 0.637, *p* < 0.001) and 0.689 (95% CI 0.671 to 0.707, *p* < 0.001) (Table 3). The cutoff value of admission SI for the prediction of all-cause mortality in the validation cohort was 0.50 with a sensitivity of 0.863 and a specificity of 0.345.

**Table 3:** Receiver operating characteristic curves of admission SI and GRACE for the prognosis prediction in the Derivation and Validation Cohorts.

	Area under ROC curve	Standard error	p-Value	95% confidence interval
Derivation Cohort				
SI	0.619	0.0314	<0.001	0.600–0.637
GRACE	0.689	0.0273	<0.001	0.671–0.707
Validation Cohort				
SI	0.672	0.0480	0.002	0.641–0.701
GRACE	0.736	0.0449	<0.001	0.707–0.764

### Prognostic Value of Shock Index in the Validation Cohort

During the 1-year follow-up, all-cause mortality in the validation cohort was 3.0% (29 cases). Univariate analysis identified multiple variables that had significant effect on all-cause mortality, including admission SI, age, LVEF, diagnosis on admission, and TIMI flow grade 3 post PCI (*p* < 0.05; Appendix S2, Table 2). After adjusting for covariates, higher admission SI continued to show significant positive correlation with the long-term all-cause mortality rate [HR = 10.091, 95% CI 2.205 to 46.187, *p* = 0.003] (Table 2).

The ROC-AUC of admission SI and GRACE score for predicting all-cause mortality in the validation cohort were 0.672 (95% CI 0.641 to 0.701, *p* = 0.002) and 0.736 (95% CI 0.707 to 0.764, *p* < 0.001) (Table 3). The cutoff value of admission SI for all-cause mortality in the validation cohort was 0.63 with a sensitivity of 0.586 and a specificity of 0.726.

### Comparison of Prognostic Value of Shock Index and GRACE Score

**Figure 1:**
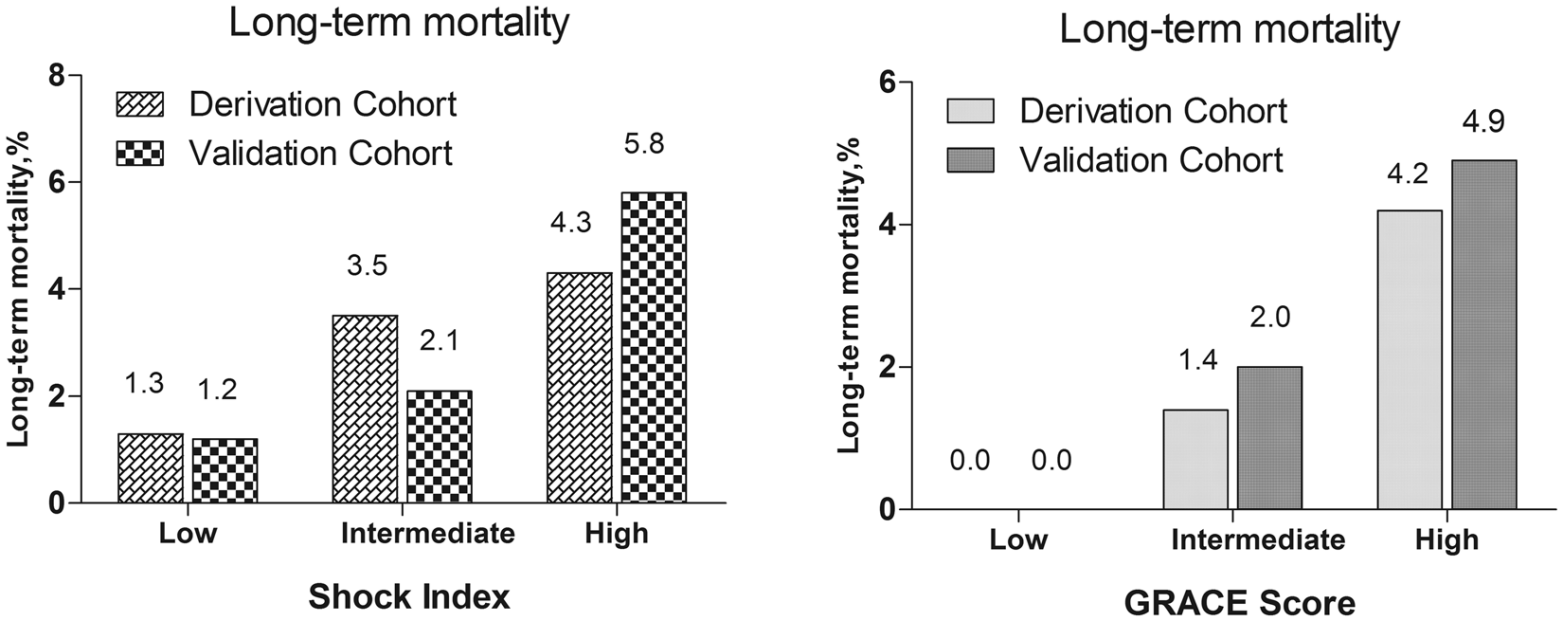
Long-term mortality in different risk stratifications according to admission SI or GRACE score in the both cohorts

**Figure 2:**
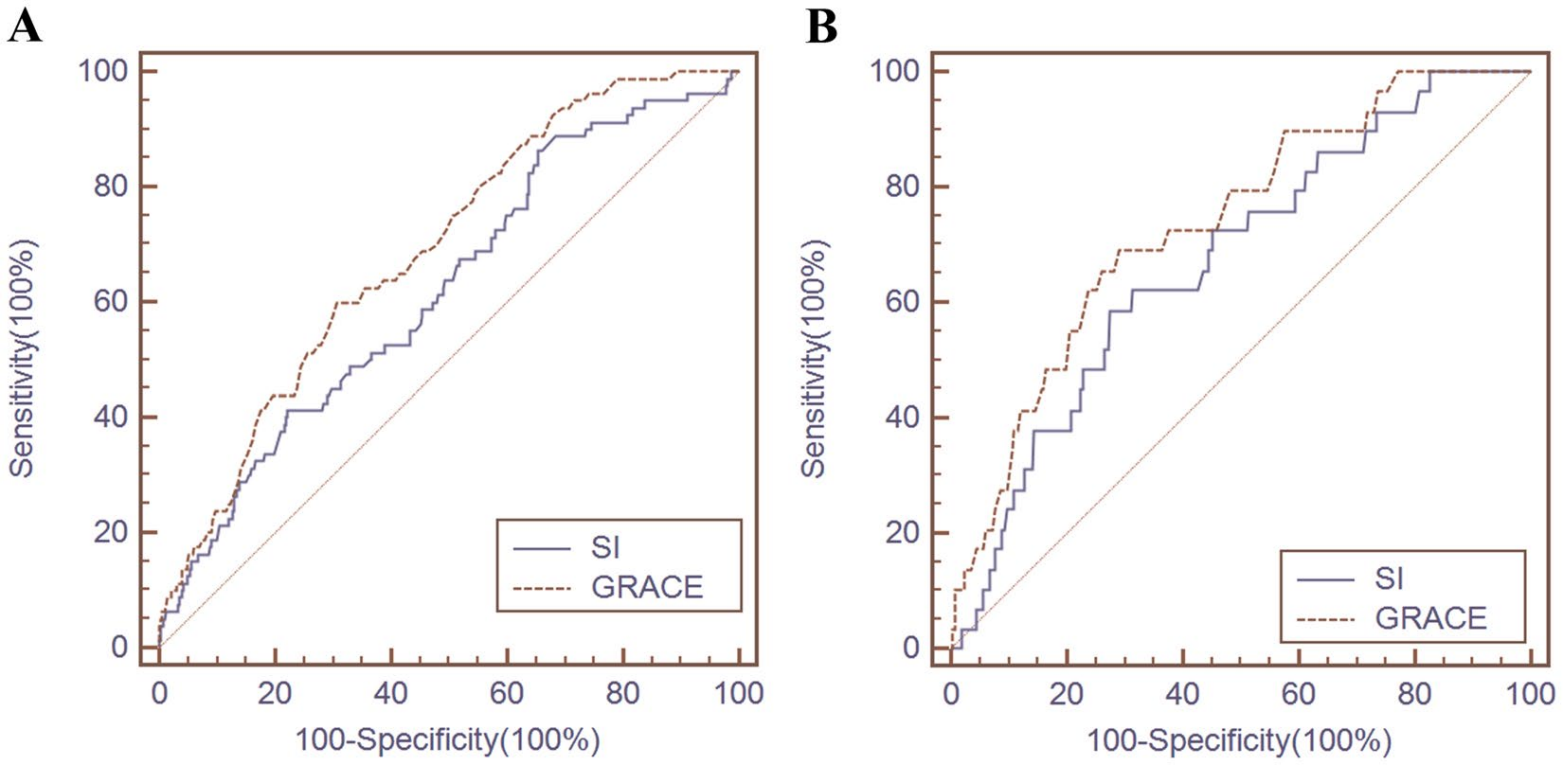
Receiver operating characteristic curves of shock index and GRACE score for all-cause mortality prediction in the derivation cohort (**A**) and the validation cohort (**B**).

Risk stratification by both admission SI and GRACE score could correctly identify high-risk patients in both cohorts (Fig. 1), and each index showed similar diagnostic performance in predicting all-cause mortality in both cohorts (admission SI *vs*. GRACE score, derivation cohort *z* = 1.919, *p* = 0.055 and validation cohort *z* = 1.039, *p* = 0.299) (Table 4 and Fig. 2A and B).

**Table 4:** Comparisons of the predictive accuracy of admission SI and GRACE for the prognosis prediction in the Derivation and Validation Cohorts.

	Difference	Z	p-Value
Derivation Cohort			
SI vs. GRACE	0.0704	1.919	0.055
Validation Cohort			
SI vs. GRACE	0.06411	1.039	0.299

## Discussion

Our study examined the predictive value of admission SI for long-term prognosis of ACS in patients undergoing PCI. The main findings of the study were: (1) high admission SI is an independent predictor of long-term mortality, and (2) the prognostic performance of admission SI was similar to that of the GRACE score for predicting long-term mortality in ACS patients undergoing PCI. In brief, we found that the risk of all-cause mortality increased by a factor of 3.104 per unit elevation of SI in the derivation cohort [HR = 4.104, 95% CI 1.553 to 10.845, *p* = 0.004] or 9.091 per unit elevation of SI in the validation cohort [HR = 10.091, 95% CI 2.205 to 46.187, *p* = 0.003] with SI as a continuous variable and, as shown in Fig. 1, SI maintained an independent and significant positive correlation with all-cause mortality as a categorical variable. Patients in the high-risk SI group had the highest all-cause mortality in both cohorts. Furthermore, our study confirmed that the prognostic value of admission SI for predicting all-cause mortality was similar to that of the GRACE score (admission SI *vs*. GRACE score: *z* = 1.919, *p* = 0.055, derivation cohort, and *z* = 1.039, *p* = 0.299, validation cohort). Accordingly, if the GRACE score is not available, SI, which can be quickly and easily obtained at the bedside, should be a useful alternative for early risk stratification after ACS in patients undergoing PCI.

The detailed pathophysiological association between SI and adverse outcome needs further evaluation. There are no definite mechanisms underlying the prognostic value of SI for long-term mortality, although there are several possible explanations for the nature of such an association. First, SI may reflect the deterioration of cardiac index, stroke volume, and LV stroke work^9^. Furthermore, patients with AMI usually suffer from overactivity of the sympathetic nervous system, which regulates heart rate and blood pressure^21^. Sympathetic hyperactivity has also been associated with the degree of LV dysfunction^21^. SI may reflect the integrated response of the cardiovascular and nervous systems. Therefore, the mechanisms that support SI as a useful predictor of long-term mortality could include the following. (1) Higher SI may reflect overactivity of the sympathetic nervous system, which is associated with fatal ventricular arrhythmias that are a common complication after AMI accounting in large part for sudden deaths among patients who survive the initial event^22^. (2) High SI is indicative of more severe cardiac dysfunction, usually followed by more extensive left ventricular remodeling and heart failure, and the latter usually causes higher mortality^23^.

SI was initially proposed by Allgöwer *et al*. as a means for assessing hemodynamic stability^8^. SI is an accurate and simple risk index for circulatory failure^9^, and subsequent studies found that it is also useful for evaluating the prognosis of critically ill patients in settings including trauma, surgery, and sepsis^10,11^. Bilkova *et al*. were the first to report on the prognostic value of SI for in patients undergoing PCI^13^, specifically, that SI was an independent predictor of in-hospital mortality in STEMI patients undergoing PCI^13^. This finding has been verified and extended by other researches, and elevated SI has now proven to be an independent predictor of adverse outcomes in STEMI^12–18^, NSTEMI^19^, and AMI^20^ patients undergoing PCI. In line with the previous studies, our study demonstrated that admission SI is an independent predictor of adverse outcome in ACS patients undergoing PCI. We also showed that admission SI has the same prognostic performance as the GRACE score for predicting all-cause mortality in ACS patients undergoing PCI. These results taken together confirm that SI can be applied in clinical practice and the immediate clinical relevance of our findings is that SI, which can easily and quickly aid with the identification of high-risk ACS patients after PCI, can be a valid adjunct or, when necessary, alternative, to the GRACE score.

This study had several limitations. First, this study was retrospective and observational, so potential confounders and selection bias could not be completely adjusted. Second, data about the patient status and medication treatment that influenced admission heart rate and/or blood pressure, such as anxiety, vagal reaction, beta-blockers and inotropes, was not complete. Third, heart rate and blood pressure were measured at only a single time point, and these data might be much different than those obtained in subsequent measurements. The calculation of SI from the mean values of heart rate and systolic blood pressure may be a more reliable method^24^. Moreover, the data, which were measured before later interventions, may be the more reliable indicator. Fourth, in this study, patients with obvious arrhythmia, such as atrial fibrillation, were excluded, because blood pressure measurement was not of good quality. However, previous research has found an association of atrial fibrillation with short- and long-term mortality among patients with AMI^25^.

## Conclusions

Admission SI was an independent predictor of adverse outcome in ACS patients undergoing PCI. Admission SI alone can identify patients at high risk of death. Admission SI was similar to the GRACE score for predicting all-cause mortality in ACS patients undergoing PCI. However, SI is easier to calculate than GRACE score.

## Methods

### Study Design and Setting

This study complies with the Declaration of Helsinki, and the Shengjing Hospital of China Medical University Research Ethics Committee approved the research protocol. Where applicable, written informed consent was formally obtained from all participants.

### Derivation Cohort

We performed this study using individuals from 2 independent cohorts. The derivation cohort (n = 2631) was recruited from a retrospective cohort whose rationale and design have been previously described^26^. In brief, from January 1, 2010 to October 31, 2014, 3007 consecutive ACS patients who were hospitalized and underwent PCI at a large-scale hospital in Northeast China (Shengjing Hospital of China Medical University, Shenyang, China) were included in the cohort. The investigators obtained clinical and procedural data for all cases from the electronic medical records, including the Picture Archiving and Communication System (PACS) interventional imaging data, and the operative reports from the PCI procedures. Left ventricular ejection fraction (LVEF) was measured during hospitalization by echocardiography and the thrombolysis in myocardial infraction (TIMI) flow grades and GRACE scores were determined as defined previously^5,27^. Exclusion criteria for the derivation cohort were: (1) atrial fibrillation or other obvious arrhythmia at blood pressure measurement (101 cases); (2) missing GRACE score (33 cases); (3) lost to follow-up (222 cases); and (4) death during the index hospitalization (20 cases). The final derivation cohort consisted of 2631 ACS patients undergoing PCI. Clinical follow-up was assessed in October 2015 by phone interviews with each patient’s general practitioner/cardiologist, the patient, or the patient’s family. All patients were followed for a mean duration of 32 months (12 to 67 months). All-cause mortality was identified in the medical records or by the referring hospital physician. All events were validated by 2 independent event-judge physicians.

### Validation Cohort

The validation cohort (n = 963) came from the ongoing, prospective, observational Prospective evaluation of prognosis of PCI patients Using network data in SHengjing Hospital of China Medical University (P-PUSH) project. P-PUSH contains comprehensive clinical and procedural data from all consecutive ACS patients receiving PCI at Shengjing Hospital of China Medical University from January 1, 2015 forward. We prepared patient care report forms (CRFs) containing 332 discrete items with subdivisions including demographic information, past history, clinical characteristics on admission, laboratory measurements, procedure-related complications, and use of cardiac medications. Participating physicians completed the CRF after the patients were discharged based on information from the electronic medical record, the PACS interventional imaging data, and the operative reports from the PCI procedure. ACS were classified by the attending cardiologists in accordance with published guidelines^1–4^. LVEF was measured by echocardiography during hospitalization and the TIMI flow grade and GRACE score were determined as defined previously^5,27^. Prospective clinical follow-up after discharge was performed regularly in all cases by direct hospital visits and telephone interviews with the patient’s general practitioner/cardiologist, the patient, or the patient’s family. All events were adjudicated and classified by 2 cardiologists. Exclusion criteria for the validation cohort were: (1) atrial fibrillation or other obvious arrhythmia that interfered with blood pressure measurement (35 cases); (2) missing GRACE (21 cases); (3) loss of follow-up (149 cases); and (4) death during the index hospitalization (17 cases). Finally, the validation cohort included 963 ACS patients undergoing PCI, all of whom had a 1-year follow-up from January 1, 2015 to January 1, 2016.

### Participants and Procedures

ACS was classified according to current guidelines^1–4^. Briefly, NSTEMI/unstable angina is defined as chest discomfort or anginal equivalent, ST-segment depression, transitory ST-segment elevation or prominent T-wave inversion, and positive/negative biomarkers (CKMB, T/I troponin). STEMI is defined as chest pain presenting < 12 h from onset of pain to time of PCI and significant ST-segment elevation ( ≥ 0.1 mV in at least 2 standard leads or ≥ 0.2 mV in at least 2 contiguous precordial leads) or new left bundle branch block. PCI was performed in accordance with current guidelines^1–4^ with aspiration thrombectomy and glycoprotein IIb/IIIa inhibitor administration performed at the discretion of the operator. The operators also prescribed periprocedural and postprocedural anti-platelet regimens and other cardiovascular medications according to the guidelines^1–4^. Admission SI is defined as the ratio of HR and SBP on admission^8,9^, and we used the first set of documented vital signs upon admission to the ward or the emergency department to calculate the admission SI in our patients. Risk stratification in both groups was based on the admission SI values from the derivation cohort as follows: first tertile, low risk, admission SI < 0.50; n = 879 in the derivation cohort and 322 in the validation cohort; second tertile, intermediate risk, admission SI 0.50 to 0.60; n = 879 in the derivation cohort and 336 in the validation cohort; third tertile, high risk, admission SI ≥ 0.61; n = 873 in the derivation cohort and 305 in the validation cohort. Risk stratification was also performed in each cohort according to GRACE scores^1–4^, as follows: low risk, GRACE score ≤ 88; n = 271, derivation cohort and 185, validation cohort; intermediate risk, GRACE score: 89 to 118, n = 691, derivation cohort and 305, validation cohort; and high risk, GRACE score > 118, n = 1669, derivation cohort and: 473, validation cohort.

### Data Availability

All data generated or analysed during this study are included in this published article and its Supplementary Information files.

### Statistical Analysis

Quantitative variables are presented as mean±standard deviation (SD) or median [interquartile range, IQR] and categorical variables are presented as counts and proportions (%). Cox proportional-hazards regression modeling by forward stepwise procedure was used to analyze the effect of variables on event-free survival. The variables that showed significance on univariate analysis (Appendix S1 and S2, *p* < 0.05) were entered into the final model (Table 2). Results are reported as hazard ratios (HRs) with associated 95% confidence intervals (CIs). The accuracy of admission SI and GRACE score for predicting all-cause mortality was assessed according to the area under the receiver operating characteristic (ROC-AUC) curve^28^ and compared via a nonparametric test developed by DeLong *et al*.^29^ with MedCalc software for Windows version 11.4.2.0 (MedCalc Software, Mariakerke, Belgium). AUC values å 0.5, 0.75, and 0.93 indicated fair, good, and very good accuracy^30^. All tests were two-sided, and statistical significance was defined as *p* < 0.05. All statistical analyses were performed with SPSS version 19 (SPSS Inc., Chicago, Illinois, USA).

## Electronic supplementary material

Supplementary Information 
