# Supplementary material for: Derivation and Validation of Shock Index as a parameter for Predicting Long-term Prognosis in Patients with Acute Coronary Syndrome
Source: Sci Rep. 2017 Sep 20;7:11929. doi: 10.1038/s41598-017-12180-2 (PMC5607331; doi:10.1038/s41598-017-12180-2)
Supplement: Supplementary file 1 — Supplementary Information [file 41598_2017_12180_MOESM1_ESM.pdf]

Derivation and Validation of Shock Index as a parameter for Predicting Long-term Prognosis in Patients with Acute Coronary Syndrome

Tongtong Yu[1], Chunyang Tian[1], Jia Song[1], Dongxu He[1], Zhijun Sun[1], Zhaoqing Sun[1]*

[1] Department of Cardiology, Shengjing Hospital of China Medical University, Shenyang, Liaoning, P.R.China

* Corresponding author:

E-mail: sunzhaoqing@vip.163.com

Tel: 86-24-9661522211

Fax: 86-24-9661522211

Appendix S1 Effects of multiple variables on all-cause mortality in Univariate Analysis in the Derivation Cohort.

	HR	95% CI	P
Age	1.056	1.035-1.078	＜0.001
Gender	1.335	0.849-2.099	0.212
History of Diabetes Mellitus	1.284	0.824-2.000	0.269
History of Hypertension	1.238	0.789-1.941	0.353
History of Dyslipidemia	0.650	0.418-1.010	0.055
Current/recent smoker	0.669	0.429-1.045	0.077
History of renal dysfunction	1.486	0.742-2.972	0.263
History of MI	2.561	1.414-4.638	0.002
Prior PCI	2.072	1.122-3.829	0.020
Prior peripheral arterial disease	3.773	1.190-11.965	0.024
SBP on admission, mm Hg	0.997	0.987-1.007	0.534
Heart rate on admission, beats/min	1.027	1.014-1.040	＜0.001
LVEF, %	0.951	0.933-0.969	＜0.001
Diagnosis on admission	1.270	0.963-1.673	0.090
Troponin-I on admission, ng/mL	1.008	1.002-1.015	0.006
Left main disease	1.346	0.694-2.612	0.379
Three-vessel disease	2.510	1.619-3.893	＜0.001
Intra-aortic Balloon Pump	5.083	2.974-8.687	＜0.001
TIMI flow grade 3 post PCI	0.137	0.034-0.557	0.005
Use of glycoprotein IIb/IIIa inhibitor	0.677	0.399-1.147	0.147
Aspirin	1.104	0.348-3.501	0.867
Clopidogrel	1.309	0.413-4.150	0.647
Ticagrelor	1.081	0.150-7.769	0.939
Statin	1.153	0.422-3.153	0.781
Angiotensin-converting enzyme inhibitors / Angiotensin receptor blockers	0.541	0.345-0.850	0.008
Beta-blockers	0.475	0.298-0.757	0.002

MI, myocardial infarction; bpm, beats per minute; LVEF, left ventricular ejection fraction; h, hour; PCI, percutaneous coronary intervention

Appendix S2 Effects of multiple variables on all-cause mortality in Univariate Analysis in the Validation Cohort.

	HR	95% CI	P
Age	1.047	1.012-1.082	0.008
Gender	1.295	0.602-2.785	0.508
History of Diabetes Mellitus	0.716	0.306-1.676	0.441
History of Hypertension	1.978	0.876-4.465	0.101
History of Dyslipidemia	1.097	0.486-2.476	0.824
Current/recent smoker	0.757	0.364-1.574	0.457
History of renal dysfunction	0.403	0.055-2.964	0.372
History of MI	0.337	0.046-2.480	0.286
Prior PCI	0.042	0.000-7.619	0.232
Prior peripheral arterial disease	0.049	0.000-9.502	0.649
SBP on admission, mm Hg	1.000	0.984-1.016	0.973
Heart rate on admission, beats/min	1.037	1.018-1.055	＜0.001
LVEF, %	0.958	0.924-0.993	0.019
Diagnosis on admission	1.490	1.033-2.150	0.033
Troponin-I on admission, ng/mL	1.005	0.994-1.015	0.375
Left main disease	1.709	0.595-4.911	0.320
Three-vessel disease	1.307	0.595-2.871	0.504
Intra-aortic Balloon Pump	2.912	0.693-12.248	0.145
TIMI flow grade 3 post PCI	0.137	0.018-1.001	0.051
Use of glycoprotein IIb/IIIa inhibitor	1.938	0.932-4.028	0.076
Aspirin	0.869	0.272-2.764	0.740
Clopidogrel	1.125	0.421-4.267	0.556
Ticagrelor	1.274	0.168-9.057	0.685
Statin	1.046	0.259-2.654	0.639
Angiotensin-converting enzyme inhibitors / Angiotensin receptor blockers	0.527	0.233-1.189	0.123
Beta-blockers	0.563	0.250-1.272	0.167

MI, myocardial infarction; bpm, beats per minute; LVEF, left ventricular ejection fraction; h, hour; PCI, percutaneous coronary intervention
